# Videofluoroscopic swallowing study predicts clinical outcomes in critically Ill children with dysphagia: a retrospective observational study

**DOI:** 10.3389/fped.2025.1507645

**Published:** 2025-02-06

**Authors:** Yoonju Na, Jaeyoung Choi, Jihong Choi, Su Mi Oh, Hyuna Jang, Suein Choi, Joongbum Cho, Jeong-Yi Kwon

**Affiliations:** ^1^ Department of Physical Medicine and Rehabilitation, Ajou University School of Medicine, Suwon, Republic of Korea; ^2^ Department of Pediatrics, Department of Critical Care Medicine, Samsung Medical Center, Sungkyunkwan University School of Medicine, Seoul, Republic of Korea; ^3^ Department of Physical and Rehabilitation Medicine, Samsung Medical Center, Sungkyunkwan University School of Medicine, Seoul, Republic of Korea; ^4^ Department of Pharmacology, College of Medicine, The Catholic University of Korea, Seoul, Republic of Korea

**Keywords:** pediatric, dysphagia, critically ill children, videofluoroscopic swallowing study (VFSS), intensive care unit (ICU)

## Abstract

**Background:**

This retrospective observational study aimed to investigate the features of acute dysphagia observed during videofluoroscopic swallowing study (VFSS) in critically ill children and their potential to anticipate clinical outcomes.

**Methods:**

Administrative healthcare data of children aged 1–18 were analyzed. Data were collected from the pediatric intensive care unit (PICU) of a single tertiary medical center in South Korea between March 2019 and December 2022. We reviewed VFSS conducted on patients in the PICU who were referred by clinicians suspecting dysphagia.

**Results:**

A total of 36 children were included in the study; 52.8% exhibited aspiration on VFSS. In this investigation, participants were provided with pureed food, liquids, solids, and a combination of solids and liquids (referred to as mixed) during the examination. Any occurrence of aspiration throughout the examination was deemed as aspiration. All individuals displaying aspiration were found to have silent aspiration. Silent aspiration was associated with a longer length of stay (LOS) in the PICU. Logistic regression analysis revealed that the time from PICU admission to VFSS and intubation duration significantly influenced LOS. Abnormal findings in the VFSS, including aspiration, delayed swallowing reflex, insufficient laryngeal closure, and residue, were statistically significant variables in determining the feeding mode at discharge.

**Conclusion:**

This study highlights the importance of VFSS in assessing swallowing function in critically ill children. It suggests that VFSS findings, such as silent aspiration, can aid in predicting patient outcomes, including LOS and the delay in oral feeding.

## 1 Introduction

Dysphagia represents a predominant concern referred to pediatric rehabilitation services from the pediatric intensive care unit (PICU), underlining its significance both as a common symptom and a potential severe complication. This condition, often observed across a spectrum of neurological disorders, may stem from a single medical issue, yet it is frequently the result of multiple comorbidities (1). In pediatric populations, feeding and swallowing difficulties are commonly reported as comorbidities in neurological diseases and in other medical conditions such as congenital heart disease (2). Among infants, prematurity is a common cause of dysphagia (3–5). Neuromuscular disorders disrupt the delicate balance of sensory and motor functions vital for secure swallowing, with delayed reflexes, hypotonia (reduced muscle tone), and generalized coordination deficits contributing significantly to the onset of dysphagia (6). Anatomic abnormalities of the upper aerodigestive tract, gastroesophageal reflux, cardiopulmonary disease could also increase the risk of difficulties with swallowing (7). Patients with these various comorbidities are prevalent in pediatric intensive care units (PICUs), yet reports on which comorbidities commonly cause dysphagia are scarce. In critically ill children, the most frequently reported cause is post-extubation dysphagia (PED) (8). The incidence rate of PED has been reported to range from 3% to 62% (9). This broad range could be due to differences in the characteristics of PICU patients, dysphagia definitions, and assessment methods. It is speculated that the incidence of dysphagia occurring in the PICU is likely higher than that of PED alone. According to a prospective observational study on PED in adult ICU conducted by Brodsky, the PED incidence rate is 18.3% (10). Notably, PED persisted until ICU discharge in most cases, and over 60% of the patients with impaired swallowing function in the ICU continued to experience difficulties in swallowing during hospital discharge. PED has a significant impact on both health and mortality, with an excessive 90-day all-cause mortality rate of 9.2% (10). In adult ICU patients, baseline neurologic disease, emergency admission, and duration of invasive mechanical ventilation appeared as prominent independent risk factors for dysphagia (11). Despite the frequency of studies on dysphagia in adult ICU settings, pediatric ICUs remain underreported. Moreover, the existing literature primarily focuses on PED, indicating a pressing need for comprehensive data on dysphagia across the entire PICU landscape.

Recently, da Silva et al. reported on PED in critically ill children (12, 13). Their results revealed a high prevalence of dysphagia, which is associated with adverse patient outcomes. It was significantly associated with longer PICU and hospital stays and a longer delay to oral feeding than in patients without dysphagia (14 vs. 7.5 days, 21 vs. 15 days, and 6 vs. 1 day, respectively) (12). Ten participants (9%) failed to resume a total oral intake at hospital discharge. They utilized a clinical evaluation tool, specifically the pediatric-adapted version of the Functional Oral Intake Scale (FOIS). No study has reported the characteristic of the VFSS results among critically ill patients in the PICU.

Therefore, this study aimed to investigate the characteristics of dysphagia occurring in PICU and to present findings from VFSS. Additionally, the study included not only PED cases but also pediatric patients who did not require mechanical ventilator support. Through this retrospective study, we aimed to identify anatomical abnormalities observed in critically ill patients and investigate whether such findings impact clinical outcomes.

## 2 Material and methods

### 2.1 Patients and study design

This study was a retrospective observational analysis. To study dysphagia in the PICU, we used administrative healthcare data from children aged 1–18 years who underwent VFSS between March 2019 and December 2022. From March 1, 2019, to December 31, 2022, a total of 1,605 pediatric patients were admitted to the PICU of our institution. Among them, 910 patients were admitted for medical (non-surgical) reasons. Our study was conducted by selecting patients from this group based on the following criteria. Children who had a tracheostomy upon admission to the PICU were excluded, as they were typically receiving tube feeding and thus considered to have dysphagia. Additionally, patients who had pre-existing dysphagia were also excluded. At our institution, clinicians refer patients for testing when considering oral feeding, especially if they exhibit clinical symptoms such as coughing or refusal to eat.

### 2.2 Ethics statement

The Institutional Review Board of Samsung Medical Center reviewed and approved the study (IRB No.2023-04-042) and waived the requirement for informed consent. This study was conducted retrospectively, and VFSS was performed as part of routine clinical decision-making by the attending physician when necessary. The consent form for VFSS included explanations regarding the physician accompanying the patient from the PICU to the examination room, the potential risks associated with the procedure, possible issues if the test was not conducted, and alternative diagnostic methods. These details were also verbally explained to the guardians to ensure full understanding, and the procedure was performed only after the guardians provided informed consent.

### 2.3 Videofluoroscopic swallowing study (VFSS)

The following describes the methodology employed in this study, where VFSS data were collected retrospectively. Examinations were conducted in accordance with established protocols at our institution.

The VFSS was performed by two experienced speech-language pathologists and a physiatrist. We used a modified version of the Logemann protocol (14). The subjects were placed in a sitting position, and swallowing was recorded from the lateral and anteroposterior views using radioactive fluoroscopic equipment (SONIALVISION G4; Shimadzu, Kyoto, Japan) in a fluoroscopy laboratory. In this study, participants were offered puree (rice porridge with finely ground rice), thin liquid (water), thick liquid (pureed pear and its juice), semi-solid (rice porridge with non-ground rice), solid (cooked rice), and mixed (cookie + milk) during examination, and any instance of aspiration at any point was considered as aspiration The examination starts with thick liquid and progresses to puree, followed by semi-solid and solid, depending on the ability to chew, or transitions directly to thin liquid if chewing is not possible. During the examination, if no aspiration occurs, the amount provided gradually increases from 1 cc or 3 cc to 5 cc and 9 cc. Position changes, such as chin tuck, are attempted for patients undergoing treatment, but in this study, consistency modification, such as adding thickener, was not attempted. In situations involving children who were only fed milk, liquid barium (Barium sulfate; Solotop sol 140, Tae Joon Pharmaceutical) was mixed with the milk. Then, this was transferred to a bottle and given to the children for testing. Liquid barium was added to the food to visualize the bolus during fluoroscopy. To minimize radiation exposure, the fluoroscopy unit was used only for the first 5–10 consecutive swallows with a frame capture rate of 30 frames per second. The maximum duration of radiation exposure was two minutes. The test was terminated if the infant presented with significant symptoms, such as desaturation, tachycardia, bradycardia, or definite tracheal aspiration during fluoroscopy.

### 2.4 VFSS interpretation

The VFSS was recorded on video and interpreted by a speech-language pathologist and a physiatrist. The video cassettes were replayed on a monitor with a video cassette recorder, allowing slow-motion and frame-by-frame analysis. In addition to verifying the presence of aspiration and penetration, we performed video analysis on the following five items: Delayed Oral Transit Time (OT), Delayed Swallowing Reflex (SR), Insufficient Epiglottic Inversion (EI), Insufficient Laryngeal Closure (LC), and Residue (R).

1. Delayed Oral Transit Time (OT): When it took more than 1.5 s for food or liquid to move from the mouth to the throat.
2. Delayed Swallowing Reflex (SR): When the swallowing action started only after food reached the area behind the tongue.
3. Insufficient Epiglottic Inversion (EI): When the epiglottis (the structure that covers the airway during swallowing) did not fully close.
4. Insufficient Laryngeal Closure (LC): When the airway did not fully seal during swallowing.
5. Residue (R): When food or liquid remained in the throat after swallowing.

Penetration-Aspiration Scale (PAS) was scored via video review by a speech-language pathologist. Penetration was noted when contrast material moved above the true vocal cords without descending further, and aspiration was identified when contrast passed below the true vocal cords. The PAS is an 8-point ordinal scale, with 1 representing the least severe and 8 representing the highest or most severe score (silent aspiration).

### 2.5 Data collection

We transcribed the data from all patients’ medical records into a custom-built computer database for the primary investigator. Patient-related data included age, sex, weight, height, time point of VFSS evaluation, PICU admission diagnostic category, disease severity at PICU admission [Pediatric Risk of Mortality (PRISM) and Pediatric Index of Mortality 3 (PIM-3) scores], and pre-existing comorbidities [complex chronic conditions (CCCs)]. Furthermore, we recorded whether intubation was performed, the reintubation within 48 h, the cumulative dose of continuous infusion drugs (vasopressors, sedatives, and neuromuscular blockers), and the length of PICU and hospital stay. We also examined the type of feeding at discharge by dividing it into alternative feeding, including nasogastric tube feeding, Percutaneous Endoscopic Gastrostomy (PEG)/Open Gastrostomy, and full oral feeding. Full oral feeding was defined as the time when the patient was well-fed with no need for gavage supplementation to maintain growth. Subgroup analyses were performed on the enrolled children, divided into two groups: patients with and without aspiration. Demographic and clinical characteristics of the two groups were analyzed and compared.

### 2.6 Statistical analysis

Categorical variables were analyzed using Pearson's chi-square test. Continuous variables were analyzed using independent sample *t*-tests and Mann-Whitney-Wilcoxon U tests to assess differences in the distribution of variables between the groups. If the data met assumptions of normality and homogeneity of variances, a two-sample *t*-test was used; otherwise, a Wilcoxon rank sum test was employed for non-normally distributed data. The associations between risk factors were analyzed using univariate and multivariate logistic regression models. We adopted a multivariable regression model using a backward selection procedure, with *P* < 0.05 as the retention criterion. Cox regression analysis was performed to identify the clinical factors that may influence the feeding mode at discharge and risk factors for aspiration. Additionally, we analyzed various clinical factors and the length of PICU and hospital stay. Covariates with *p* < 0.05 in univariate analysis were entered into the multivariable model. *P* values <0.05 were considered statistically significant. All statistical analyses were performed using the R software (version 3.6.3; Foundation for Statistical Computing, Vienna, Austria).

## 3 Result

### 3.1 Patients’ demographic and clinical characteristics

Out of the 910 patients admitted for medical reasons during the study period, 36 met the study criteria, accounting for approximately 4% of the total patient population.A total of 36 children were included in the analysis (Table 1), of whom 19 (52.8%) were boys, and 17 (47.2%) were girls, with a median age of 9.0 years [interquartile range (IQR): 3.0–14.0 years]. In our study, admission diagnoses were collected as part of the demographic characteristics. Specifically, 1 patient was admitted for cardiac reasons, 16 for respiratory issues, 10 for neurologic conditions, and 9 for other reasons, including hemato-oncology and infection/inflammation. Among 36 patients, 19 patients (52.8%) showed aspiration at any consistency in the VFSS findings, and 17 patients (47.2%) had no aspiration. In our study, of the 19 patients, 15 demonstrated aspiration with thin liquids, 7 with thick liquids, 6 with puree, and 4 with semi-solids, indicating varying levels of swallowing difficulties across different consistencies (Supplementary Table 1). Among the total of 19 patients, aspiration was observed in 6 patients during puree, 4 patients during semi-solids, 7 patients during thick liquid, and 17 patients during thin liquid. In the group with aspiration, there was a significant difference in intubation status (*p* = 0.018) and intubation duration (*p* = 0.009) compared to the group without aspiration. In the aspiration group, 84% of patients were intubated, and the duration of intubation was significantly longer, with a median of 221 h. In contrast, the no-aspiration group had a intubation rate of 47% and a median duration of 0 h. The reasons for admission to the PICU were statistically significant between the two groups (*p* = 0.009), with respiratory causes being the most common. Both groups showed no significant difference in disease severity as measured by PRISM and PIM-3 scores (*p* = 0.76 and *p* = 0.68, respectively). Additionally, there was no significant difference in the number of complex chronic conditions (CCC) between the two groups (*p* = 0.40). Silent aspiration predicted a longer length of stay (LOS) in the PICU (16 days longer, *p*-value = 0.022); however, there was no significant differences in hospital LOS. Fourteen (74%) of children with silent aspiration failed to resume total oral intake at hospital discharge, whereas 15 (88%) children in the no-aspiration group achieved oral feeding at discharge (*p* = 0.022).

**Table 1:** Demographic characteristics.

Characteristics	Overall (*N* = 36)	No aspiration group (*N* = 17)	Aspiration group (*N* = 19)	*P* values
Age (year), median (IQR)	9.0 (3.0–14.0)	11.0 (4.0–15.0)	7.0 (3.0–14.0)	0.40
The time from PICU admission to VFSS (days)	8 (5–24)	5 (5–14)	14 (8–34)	0.06
Weight (kg), median (IQR)	27 (12–48)	40 (15–49)	23 (11–39)	0.26
BMI	17.5 (15.4–18.9)	17.9 (16.9–19.8)	17.5 (13.5–18.8)	0.22
Height (cm), median (IQR)	133 (88–157)	146 (99–157)	114 (85–152)	0.36
Intubation, *n* (%)	24 (67)	8 (47)	16 (84)	0.018*
Duration of intubation (h), median (IQR)	66 (0–300)	0 (0–74)	221 (22–386)	0.005**
PICU stay (day), median (IQR)	18 (11–36)	13 (9–18)	29 (14–46)	0.022*
Hospital stay (day), median (IQR)	32 (17–71)	28 (17–59)	49 (22–77)	0.55
Sex				0.52
Female	17 (47)	9 (53)	8 (42)	
Male	19 (53)	8 (47)	11 (58)	
Reason for PICU admission				0.009**
Cardiac	1 (2.8)	0 (0)	1 (5.3)	
Neurologic	10 (28)	5 (29)	5 (26)	
Respiratory	16 (44)	4 (24)	12 (63)	
Others	9 (25)	8 (47)	1 (5.3)	
Disease severity at admission				
PRISM, median (IQR)	8 (2–13)	11 (5–18)	5 (0–9)	0.076
PIM-3, median (IQR)	−2.65 (−3.61 to −1.80)	−2.31 (−3.58 to −1.81)	−3.02 (−3.53 to −1.91)	0.68
CCC, median (IQR)	1.00 (0.00–1.00)	1.00 (0.00–1.00)	1.00 (0.00–1.00)	0.40
Continuous infusion drugs				
Midazolam, *n* (%)	25 (69)	11 (65)	14 (74)	0.56
Fentanyl, *n* (%)	28 (78)	14 (82)	14 (74)	0.70
Ketamine, *n* (%)	20 (56)	10 (59)	10 (53)	0.71
Dexmetdetomidine, *n* (%)	5 (14)	2 (12)	3 (16)	>0.99
NMBA, *n* (%)	11 (31)	5 (29)	6 (32)	0.89
Feeding mode at discharge to home, *n* (%)				<0.001***
Alternative	16 (44)	2 (12)	14 (74)	
Oral	20 (56)	15 (88)	5 (26)	
VFSS finding (any), *n* (%)	27 (75)	8 (47)	19 (100)	<0.001***

The aspiration group included patients with Penetration Aspiration Scale (PAS) score >6. VFSS, video fluoroscopic swallowing study; BMI, body mass index; PICU, pediatric intensive care unit; PRISM, pediatric risk of mortality; PIM-3, pediatric index of mortality 3; CCC, complex chronic condition; NMBA, neuromuscular blocking agent.

* *P* < 0.05.

** *P* < 0.01.

*** *P* < 0.001.

### 3.2 VFSS findings and PAS score

Patients with aspiration showed abnormal findings, including delayed swallowing reflex (SR), insufficient epiglottic inversion, insufficient laryngeal closure, and residue in the pharynx, all of which were significantly different between the groups (*p* < 0.001, *p* = 0.003, *p* < 0.001, and *p* < 0.001, respectively) (Supplementary Table 2). Among the 36 patients, 8 showed only penetration, and 19 showed aspiration. Furthermore, all 19 children (100%) with aspiration showed silent aspiration (PAS 8) (Supplementary Table 3).

### 3.3 Clinical factors associated with silent aspiration and feeding mode at discharge

Univariate analysis (Table 2) revealed that intubation (*p* = 0.030, OR = 5.62) and intubation duration (*p* = 0.023, OR = 1.01) significantly increased the risk of silent aspiration. In the VFSS findings, EI (*p* = 0.0006, OR =  9.17), LC (*p* = 0.0005, OR = 24.3), and R (*p* = 0.0003, OR = 31.2) were significantly associated with silent aspiration. Other factors such as age, sex, weight, height, and various medications (including midazolam, fentanyl, ketamine, dexmedetomidine, and neuromuscular blocking agents) did not show statistically significant associations with aspiration. Additionally, the initial severity of illness, as measured by Pediatric Risk of Mortality (PRISM) and Pediatric Index of Mortality 3 (PIM-3), was not statistically associated with the occurrence of aspiration.

**Table 2:** Clinical factors affecting aspiration.

	Univariate		Multivariate	
	Crude OR(95% CI)	*P* value	Adjusted OR (95% CI)	*P* value
Intubation	5.62 (1.28–31.3)	0.030*	2.53 (0.23–37.1)	0.44
Intubation duration (hour)	1.01 (1.00–1.02)	0.023*	1.00 (1.00–1.01)	0.31
PICU stay	1.05 (1.01–1.11)	0.045*	1.01 (0.96–1.07)	0.76
Hospital stay	1.01 (0.99–1.02)	0.54		

The aspiration group included patients with a Penetration Aspiration Scale (PAS) score >6. PICU, Pediatric Intensive Care Unit.

* *P* < 0.05.

Factors such as the severity of illness (PIM-3 and PRSIM) or intubation were not statistically significant in determining the feeding mode at discharge, which was full oral intake. Other clinical factors, including age, sex, weight, height, intubation status, and the use of medications such as midazolam, fentanyl, ketamine, dexmedetomidine, and neuromuscular blocking agents, showed no statistically significant associations with the feeding mode in the univariate analysis. However, abnormal VFSS findings that hindered full oral intake, including aspiration (*p* < 0.001, OR = 0.04), delayed SR (*p* = 0.035, OR = 0.09), insufficient laryngeal closure (*p* = 0.037, OR = 0.16) and R (*p* = 0.020, OR = 0.13), were identified as statistically significant variables in the univariate analysis (Table 3).

**Table 3:** Clinical factors affecting feeding mode (full oral intake) at discharge.

	Univariate		Multivariate	
	Crude OR (95% CI)	*P* value	Adjusted OR (95% CI)	*P* value
Videofluoroscopic study finding				
Aspiration	0.04 (0.00–0.20)	<0.001***	0.08 (0.01–0.51)	0.006**
Delayed Oral transit Time (OT)	0.56 (0.10–3.01)	0.50		
Delayed swallowing reflex (SR)	0.09 (0.00– 0.60)	0.035*	0.62 (0.02–32.3)	0.80
Insufficient epiglottic inversion (EI)	0.30 (0.06–1.21)	0.10		
Insufficient laryngeal closure (LC)	0.16 (0.02–0.78)	0.037*	0.86 (0.01–230)	0.95
Residue (R)	0.13 (0.02–0.63)	0.020*	1.33 (0.01–44.7)	0.88

The aspiration group included patients with a Penetration Aspiration Scale (PAS) score >6. BMI, VFSS, video fluoroscopic swallowing study.

* *P* < 0.05.

** *P* < 0.01.

*** *P* < 0.001.

We conducted an additional logistic regression analysis to examine factors affecting PICU and hospital LOS. In the univariate analysis, BMI, time from PICU admission to VFSS, intubation status, ketamine use, and abnormal VFSS findings (aspiration and residue in the vallecular fossa and pyriform sinus) were significant factors affecting PICU LOS (Table 4). In the multivariate analysis, time to PICU admission to VFSS (*p* = 0.037, OR =  0.12) was significantly associated with PICU LOS. It was found that late timing of VFSS (*p* = 0.002, OR = 1.5) and longer intubation duration (*p* = 0.022, OR =  0.07) were significant factors that contributed to a longer length of hospital stay (Table 5). However, in the subsequent multivariate analysis, these factors were not found to be significant in explaining hospital LOS. Additionally, factors such as the severity of illness, measured by PIM-3 and PRISM scores, the initial reason for admission (e.g., respiratory, cardiac, or others), the use of sedation or neuromuscular blocking agents (NMBA) during the hospital stay, and various VFSS findings (e.g., the odds ratio for cases with aspiration) were analyzed. However, no statistically significant associations with hospital LOS were identified.

**Table 4:** Clinical factors affecting PICU length of stay.

	Univariate		Multivariate	
	Crude OR (95% CI)	*P* value	Adjusted OR (95% CI)	*P* value
Age (year)	0.19 (−0.87–1.2)	0.72		
Sex, male	8.4 (−3.2–20)	0.15		
Weight (kg)	0.15 (−0.16–0.45)	0.33		
Height (cm)	0.01 (−0.16–0.17)	0.95		
BMI	1.8 (0.23–3.4)	0.026*	0.80 (−0.57–2.2)	0.24
Time of VFSS evaluation (day)	0.85 (0.53–1.2)	<0.001***	0.45 (0.02–0.88)	0.043*
Intubation	7.8 (−4.6–20)	0.21		
Intubation duration (hour)	0.03 (0.01–0.06)	0.011*	−0.01 (−0.04–0.01)	0.37
Reason for PICU admission				
Respiratory	−2.0 (−17–13)	0.78		
PIM-3	2.7 (−1.3–6.7)	0.18		
PRISM	0.19 (−0.58–0.96)	0.62		
CCC	−0.76 (−8.3–6.8)	0.84		
Medication				
Midazolam	5.4 (−7.4–18)	0.40		
Fentanyl	4.9 (−9.4–19)	0.49		
Ketamine	16 (5.2–27)	0.005**	7.1 (−2.3–17)	0.13
Dexmedetomidine	6.3 (−12–25)	0.50		
NMBA	3.3 (−9.9–17)	0.61		
Feeding mode at discharge				
Oral	−6.0 (−18–5.9)	0.31		
VFSS finding				
Aspiration	12 (1.1–24)	0.032*	7.7 (−4.9–20)	0.22
Delayed Oral transit Time (OT)	−1.9 (−17–13)	0.80		
Delayed swallowing reflex (SR)	9.4 (−4.0–23)	0.16		
Insufficient epiglottic inversion (EI)	5.9 (−6.2–18)	0.33		
Insufficient laryngeal closure (LC)	12 (−0.18–24)	0.053		
Residue (R)	14 (2.4–26)	0.020*		

BMI, body mass index; VFSS, video fluoroscopic swallowing study; PICU, pediatric intensive care unit; PRISM, pediatric risk of mortality; PIM-3, pediatric index of mortality 3; CCC, complex chronic condition; NMBA, neuromuscular blocking agent.

* *P* < 0.05.

** *P* < 0.01.

*** *P* < 0.001.

**Table 5:** Clinical factors affecting total hospital length of stay.

	Univariate		Multivariate	
	Crude OR (95% CI)	*P* value	Adjusted OR (95% CI)	*P* value
Age (year)	−0.42 (−3.1–2.2)	0.75		
Sex, Male	−12 (−41–18)	0.43		
Weight (kg)	0.01 (−0.76–0.78)	0.98		
Height (cm)	−0.13 (−0.53–0.28)	0.53		
BMI	3.5 (−0.51–7.6)	0.085		
Time of VFSS evaluation (day)	1.5 (0.59–2.5)	0.002**	0.51 (−0.90–1.9)	0.47
Intubation	5.1 (−26–37)	0.74		
Intubation duration (hour)	0.07 (0.01–0.14)	0.022*	0.03 (−0.04–0.10)	0.47

BMI, body mass index; VFSS, video fluoroscopic swallowing study.

* *P* < 0.05.

** *P* < 0.01.

## 4 Discussion

This study provides significant insights into the prevalence and implications of silent aspiration in children within the PICU setting, presenting detailed findings from VFSS. We observed that silent aspiration is notably prevalent among critically ill children, a condition characterized by the passage of food or liquid into the airway below the true vocal cords without eliciting a cough reflex. Children are more likely to aspirate silently than adults, which may be due to immature neurological development or the increased survival rate in premature babies and children with complex medical disorders (15).

Comparing our findings to existing literature, Velayutham et al. (16) reported a significant incidence of silent aspiration among children undergoing VFSS, with a predominant occurrence in those who aspirated thin fluids. They found that among 1,286 patients under the age of 18 who underwent VFSS in a tertiary children's hospital, 440 (34.2%) demonstrated aspiration, with 393 (89.3%) of these cases being silent aspiration (16). The factor associated with silent aspiration were several underlying conditions. The most significant predictors of silent aspiration are age, laryngeal cleft, laryngomalacia, unilateral vocal fold paralysis, developmental delay, epilepsy/seizures, syndromes, and cardiac disease (15), which are common diagnoses in children in PICU. Our research builds on these findings, identifying intubation duration and the act of intubation itself as significant predictors of aspiration risk. This aligns with the broader spectrum of dysphagia risk factors identified in ICU settings, including central nervous system impacts, peripheral nerve or muscular diseases, and the adverse effects of prolonged intubation on swallowing mechanics. Zuercher et al. (8) outlined various causes of dysphagia in the ICU. Dysphagia occurring in the ICU can be caused by (1) the central nervous system (e.g., stroke); (2) peripheral nerve damage or primary muscular disease (e.g., inflammatory myopathies); (3) medication or toxic/drug side-effects; (4) direct trauma caused by endotracheal and tracheostomy tubes; (5) neuromyopathy resulting in muscular weakness, (6) diminished laryngeal sensory function; (7) gastroesophageal reflux; and (8) dyssynchronous breathing and swallowing. The endotracheal tube prevents normal swallowing and reduces the passive opening of the upper esophageal sphincter, interfering with rapid esophageal clearance. According to Sue et al. (17), prolonged intubation is also known to cause arytenoid dislocation or subluxation, leading to obstructive closure during swallowing. Interestingly, our study found that the use of long-term sedatives and neuromuscular blocking agents was not strongly associated with swallowing dysfunction, suggesting a low likelihood of muscular weakness as a contributing factor.

Moreover, silent aspiration predicted a longer PICU LOS and poor oral feeding outcomes at hospital discharge. This finding not only aligns with but also builds upon the findings of da Silva et al. (12), who reported extended hospital and PICU stays and delayed oral feeding in children with dysphagia. Our research underscores the critical role of early detection and intervention in managing dysphagia within the PICU. Early identification of silent aspiration through VFSS can facilitate and timely therapeutic interventions, potentially reducing the PICU length of stay and improving oral feeding outcomes at discharge. Dysphagia in the PICU often increases hospitalization length and remains unresolved until discharge or beyond. By addressing swallowing difficulties early and implementing appropriate interventions, there is a possibility of reducing complications associated with aspiration, potentially leading to shorter PICU stays. Considering the high proportion of PICU patients admitted with respiratory conditions, further studies are needed to investigate how early detection of aspiration impacts clinical outcomes. Da Silva et al. reported that dysphagia was significantly associated with longer PICU and hospital stays and a longer delay to oral feeding than patients without dysphagia (14 vs. 7.5 days, 21 vs. 15 days, and 6 vs. 1 day, respectively) (12). Children with acute dysphagia diagnosed or developed after PICU admission in our study showed a much longer LOS (29 days in the PICU and 49 days in the hospital). The discrepancies between the studies are likely to be due to differences in inclusion criteria. This study included patients with clinically suspected aspirations referred for VFSS. The severity of the dysphagia in this study may be much higher than reported in the previous study by da Silva et al. (12, 13). Reducing ICU LOS is known to have a significant impact not only on clinical outcomes, such as patient mortality, but also on reducing the medical burden (18, 19). Therefore, in cases when dysphagia is suspected, it is advisable for healthcare providers to assess the need for swallowing evaluations, including the VFSS, at the time of discharging patients to a ward or home, depending on clinical indications. Clinicians should also understand that the absence of a cough does not rule out the possibility of aspiration. Moreover, identifying potential swallowing difficulties in children at high risk for dysphagia underscores the importance of initiating swallowing therapy interventions early, ideally starting in the PICU setting. Research in this area is essential to quantitatively assess the benefits of integrating swallowing therapy into the early stages of patient care within the PICU.

Our study broadens the scope by including patients beyond those with PED, offering a more comprehensive view of dysphagia in pediatric critical care. Dysphagia in children is important because it is directly related to growth and development (20–22). In addition, dysphagia leads to malnutrition and growth failure and causes significant stress for children as well as parents; therefore, active early intervention in the PICU is required (8). Da Silva et al. (12) found that PED patients were younger and had a significantly higher severity of illness score (PRISM-II) compared to non-dysphagia patients. However, our study results did not show any significant associations between age, illness severity, and dysphagia. This suggests that when we expand our analysis to include a group of non-intubated patients, it highlights the importance of considering VFSS evaluation regarding the presence of dysphagia.

Given that longer intubation periods and delayed VFSS evaluations were associated with prolonged PICU stays, it would be advisable for physicians to consider developing a protocol for performing a dysphagia work-up if intubation is prolonged. In the group with aspiration, the time to VFSS evaluation was delayed by approximately 6 days compared to the group without aspiration (14 days vs. 8 days), suggesting that such delays in evaluation could be associated with longer PICU stays. Furthermore, in this study, all cases of aspiration were silent aspiration without symptoms, suggesting that it may be worthwhile to consider screening even in patients who were not intubated.

The evaluation of children with feeding disorders is not standardized (7, 23). VFSS is the most commonly used test to evaluate patients with dysphagia and provides a fluoroscopic view of the swallow while the children are fed barium at different consistencies. During the VFSS, the children were positioned as close as possible to their regular feeding position, and all four phases of swallowing are assessed using a lateral radiographic view. This procedure serves as the sole objective measure for confirming penetration and aspiration. Adhering to the As Low As Reasonably Achievable (ALARA) principle is crucial to ensuring that VFSS is both safe and effective, using the minimum radiation dose necessary for accurate diagnosis. In our study, we limited exposure time to under 5 min and made efforts to minimize unnecessary field of view. However, radiation exposure remains an inherent limitation of the VFSS procedure itself. Nevertheless, a limitation of this procedure is that it cannot be performed in children who are unable to travel to the radiology department or maintain a sufficient sitting position for the examination. VFSS may help prevent unnecessary increases in LOS and readmission due to aspiration pneumonia. Furthermore, VFSS can provide valuable information on the best food texture to use, recommendations of tube feeding, the use of thickner and best method (e.g., chin tuck, head rotation, etc.) for safe swallowing. Another commonly used test for diagnosing swallowing disorders is Fiberoptic Endoscopic Evaluation of Swallowing (FEES). FEES has gained prominence as a substitute for VFSS. FEES provides a direct view of the airway during actual feeding without radiation exposure (24). FEES can be performed with a movable device that is easily carried to the PICU. However, FEES has disadvantages, including a temporary whiteout period at the pharyngo-esophageal transfer (25) and the inability to detect micro-aspiration (3, 7, 26). As our study revealed, it is crucial for physicians in the PICU to promptly suspect dysphagia in patients and expedite the timing of evaluations. Therefore, it may be necessary to consider using FEES as part of a screening protocol in the ICU.

Our study has several strengths compared to previous research on PED. By integrating our findings with existing literature, we highlight the necessity of incorporating VFSS into the standard care protocol for critically ill children suspected of having dysphagia. This integration not only facilitates the early detection of silent aspiration but also informs tailored intervention strategies to mitigate the adverse outcomes associated with dysphagia in this vulnerable population. Our study emphasizes the importance of a multidisciplinary approach to dysphagia management in the PICU, suggesting that timely VFSS evaluations should be considered for patients showing clinical signs of swallowing difficulties, regardless of their intubation status. One of strengths is that we included a wider range of patients in the intensive care unit suspected of having dysphagia, including a group that did not require ventilators. Building upon the study by da Silva et al. (12), which utilized the FOIS scale, our research, defined by VFSS findings, offers complementary insights into anatomical abnormalities and information on the viable, safe consistencies for feeding, thus enhancing dysphagia management in PICU patients. Since all patients were diagnosed with dysphagia by VFSS finding, we included patients with both mild and clinically significant dysphagia in our study. This provides a comprehensive understanding of dysphagia within the overall PICU population.

Additionally, our study used an objective tool, VFSS, to provide specific characteristics of VFSS findings in the ICU population. This allowed us to demonstrate that performing the test early can lead to a shorter length of stay in the ICU for patients, which could be considered one way to reduce medical costs.

This study has several limitations. First, it was conducted at a single tertiary hospital in South Korea, which may limit the applicability of our results to other settings. Another limitation of our study was the small sample size, which may have affected the generalizability of our findings. In addition, this study excluded patients who had already been diagnosed with dysphagia. Patients receiving both oral and tube feeding simultaneously were also excluded. Therefore, it is a limitation that the study participants cannot be said to represent all patients in PICU. The COVID-19 pandemic resulted in a relatively reduced number of in-hospital intensive care unit patients, which may have impacted the statistical power of our analysis. In this study, VFSS examinations were conducted when deemed necessary by the clinical physician. Therefore, our study is limited by the lack of a clinically structured assessment tool to suspect aspiration.

Despite the limitations of this study, these findings have important clinical implications. First and foremost, it is essential to evaluate patients when dysphagia is suspected in PICU. Our research revealed a correlation between VFSS findings and extended lengths of stay in PICUs. Notably, even considering the diversity of the pediatric population admitted to the ICU, factors such as age and disease severity were found to have little correlation with the mode of feeding at the time of discharge. This finding is noteworthy for clinical practice. For future research, it is crucial to explore not only VFSS but also other portable dysphagia evaluation methods, such as Fiberoptic Endoscopic Evaluation of Swallowing (FEES), in the ICU setting. Investigating these alternative approaches and their clinical outcomes in a larger ICU patient population would provide valuable insights into optimizing patient care.

## 5 Conclusions

In conclusion, our study provides valuable contributions to the current knowledge base by elucidating the patterns of dysphagia in critically ill children and the predictive value of VFSS in identifying silent aspiration. Our study highlights the critical importance of using VFSS to assess dysphagia in critically ill children. It also points out the usefulness of VFSS in predicting significant patient outcomes, such as the length of stay in PICU and delays in initiating oral feeding. These insights are crucial for improving the quality of care for these vulnerable young patients. Early identification and intervention of silent aspiration using VFSS in critically ill children with acute dysphagia may help improved clinical outcomes and underscores the need for further research to explore comprehensive dysphagia management strategies in pediatric intensive care settings.

## Data Availability

The original contributions presented in the study are included in the article/Supplementary Material, further inquiries can be directed to the corresponding author.
