# Supplementary material for: Videofluoroscopic swallowing study predicts clinical outcomes in critically Ill children with dysphagia: a retrospective observational study
Source: Front Pediatr. 2025 Feb 6;13:1507645. doi: 10.3389/fped.2025.1507645 (PMC11839649; doi:10.3389/fped.2025.1507645)
Supplement: Supplementary file 1 [file Datasheet1.docx]

Supplementary Material

**Supplementary Table 1. Incidence of Aspiration by Food Consistency in 19 patients who confirmed to have aspiration on VFSS**

DP: Dysphagia patient ; Thin L: Thin liquid; Thick L: Thick liquid

**Supplementary Table 2. Video fluoroscopic swallowing study (VFSS) findings**

| Description | Overall (N=36) | No Aspiration  (N=17) | Aspiration  (N=19) | | P values |
| --- | --- | --- | --- | --- | --- |
| Delayed Oral transit Time (OT) , n(%) | 7 (19) | 2 (12) | | 5 (26) | 0.41 |
| Delayed swallowing reflex (SR) , n(%) | 27 (75) | 8 (47) | 19 (100) | | <0.001*** |
| Insufficient epiglottic inversion(EI) , n(%) | 22 (61) | 6 (35) | 16 (84) | | 0.003** |
| Insufficient laryngeal closure(LC) , n(%) | 25 (69) | 7 (41) | 18 (95) | | <0.001*** |
| Residue(R) , n(%) | 24 (67) | 6 (35) | 18 (95) | | <0.001*** |

The aspiration group included patients with Penetration Aspiration Scale (PAS) score >6. *p<0.05, **p<0.01, ***p<0.001

**Supplementary Table 3. PAS score distribution.**

| Characteristic | n(%) |
| --- | --- |
| No penetration (PAS 1,2) | 9(25) |
| Penetration only (PAS 3,4 5) | 8(22) |
| Aspiration (PAS 8) | 19(53) |
| PAS score, Mean ±SD | 5.42 ± 3.03 |

PAS: Penetration Aspiration Scale
